# Concentration of Lymph Node Aspirate Improves the Sensitivity of Acid Fast Smear Microscopy for the Diagnosis of Tuberculous Lymphadenitis in Jimma, Southwest Ethiopia

**DOI:** 10.1371/journal.pone.0106726

**Published:** 2014-09-03

**Authors:** Mulualem Tadesse, Gemeda Abebe, Ketema Abdissa, Alemayehu Bekele, Mesele Bezabih, Ludwig Apers, Robert Colebunders, Leen Rigouts

**Affiliations:** 1 Department of Medical Laboratory Sciences and Pathology, Jimma University, Jimma, Ethiopia; 2 Department of Clinical Sciences, Institute of Tropical Medicine, Antwerp, Belgium; 3 Department of Epidemiology and Social Medicine, University of Antwerp, Antwerp, Belgium; 4 Mycobacteriology Unit, Department of Microbiology, Institute of Tropical Medicine, Antwerp, Belgium; Fundació Institut d’Investigació en Ciències de la Salut Germans Trias i Pujol. Universitat Autònoma de Barcelona. CIBERES, Spain

## Abstract

**Background:**

Tuberculous lymphadenitis (TBLN) is the most common form of extrapulmonary tuberculosis. The cytomorphological features of lymph node smears have reduced specificity for the diagnosis of tuberculosis. The diagnosis of TBLN with direct smear microscopy lacks sensitivity due to the limited number of bacilli in lymph node aspirate. Therefore, we aimed to assess whether the concentration of lymph node aspirate improves the sensitivity of acid fast smear microscopy for the diagnosis of tuberculous lymphadenitis.

**Methods:**

A cross-sectional comparative study was conducted on 200 patients clinically suspected for tuberculous lymphadenitis in Jimma, Ethiopia. Lymph node aspirate was collected. The first two drops were used for cytomorphological study and direct acid fast staining. The remaining aspirate was treated with N-acetyl-L-cysteine (NALC) and concentrated by centrifugation at 3000 g for 15 minutes. The sediment was used for acid fast staining and culture. Differentiation of *M. tuberculosis* complex (MTBC) from non-tuberculous mycobacteria (NTM) was done by para-nitrobenzoic acid susceptibility test.

**Result:**

Complete data were available for 187 study subjects. 68% (127/187) were positive for *M. tuberculosis* on culture. Four isolates, 2.1% (4/187), were identified as NTM. The detection rate of direct smear microscopy was 25.1% and that of the concentration method 49.7%. Cytomorphologically, 79.7% of cases were classified as TBLN. The sensitivity of direct smear microscopy was 34.6%, for concentrated smear microscopy 66.1%, and for cytomorphology 89.8%. Two AFB positive cases on concentration method were non-tuberculosis mycobacteria (NTM). The concentration method yielded a positive result from seven cases diagnosed as suppurative abscess by cytology. Both for the direct and concentration methods the highest rate of AFB positivity was observed in smears showing caseous necrosis alone. Smear positivity rate decreased with the appearance of epithelioid cell aggregates.

**Conclusion:**

The concentration of lymph node aspirates for acid fast smear microscopy had significantly higher sensitivity than direct microscopy.

## Background

Tuberculosis (TB) is a global health burden especially in the developing world [1]. Ethiopia ranks 8^th^ among the 22 high TB burden countries and third in terms of the number of extrapulmonary tuberculosis (EPTB) cases. The prevalence of all forms of TB is estimated at 261 per 100,000 population, leading to an annual mortality rate of 64 per 100 000 [2].

In Ethiopia, EPTB accounted for 34.8% of TB cases with the largest group being tuberculous lymphadenitis (80%) [3]. Definitive diagnosis of tuberculous lymphadenitis is often difficult as most of the available techniques are low either in sensitivity or specificity as compared to culture. Clinical features, though indicative of tuberculous etiology, are not adequate for making a definitive diagnosis [4]. Fine needle aspiration cytology (FNAC) is a simple and rapid diagnostic technique but is characterised by low specificity [5]. Direct smear microscopy is the cornerstone for diagnosis of pulmonary TB in developing countries. It is rapid, inexpensive, specific, and capable of identifying the most infectious cases of TB, but its sensitivity in EPTB cases is limited to 20–43% [6], [7], [8]. Mycobacterial culture is the gold standard method for detection of tubercle bacilli with the sensitivity ranging from 70% to 80% [7], but it is time-consuming and requires specialized safety procedures as it is performed in a biosafety level-2 facility.

Studies have shown that the sensitivity of smear microscopy can be improved if the sputum sample is liquefied with one or more chemical reagents and then concentrated by centrifugation before acid fast staining [6], [7], [8], [9]. N-acetyl-L cysteine (NALC) is used for the treatment of sputum for culture. This study aimed to assess whether treatment with NALC followed by concentration improves the sensitivity of smear microscopy of lymph node aspirate. The basic assumption was that the NALC powder in this solution liquefies the specimen and lyses the cells, including the ones containing the bacilli, after which the solution can be further concentrated by centrifugation. To the best of our knowledge no study so far has evaluated a concentration method to detect mycobacterial bacilli in lymph node aspirates in Ethiopia.

## Methods

### Recruitment of study participants

A cross-sectional comparative study was conducted at Jimma University Specialized Hospital from April1, 2012 to September 30, 2012. A total of 200 clients with suspected TBLN and subjected to fine needle aspirate were included in the study. Patients on anti-tuberculosis treatment at the time of the lymph node aspiration were excluded from the study. Demographic and clinical information of the participants was collected by trained clinical nurses using a pre-tested questionnaire. HIV test results were collected from the medical records after obtaining written consent from the clients.

### Collection of lymph node aspirate

Lymph node aspirate was collected after receiving written consent from the study participants. Briefly FNA was performed from a swollen superficial lymph node by using a sterile 21-gauge needle with an attached syringe. The overlying area was cleaned with 70% alcohol. Then the node was punctured by developing a negative pressure in the syringe. Multiple (average six) in and out passes were made by the needle without exiting the node. After removing the needle drops of aspirate were placed on two clean slides for FNA cytology and direct AFB smear. The rest of the specimen was transferred into a falcon tube containing sterile normal saline for concentrated ZN staining and culture at Jimma University Laboratory of Mycobacteriology.

The gross appearance of aspirate was noted as caseous for cheese like or yellow-white aspirate and purulent/non-caseous for greenish yellow or yellow aspirate.

### Fine needle aspiration cytology (FNAC)

FNA smears were prepared on clean slides on the spot. The slides were air dried and flooded with freshly filtered Wright’s stain and buffered with clean tap water. The buffered slides were continuously stained with Wright’s stain for 10 minutes, washed with tap water and air dried. Finally, the slides were examined by a pathologist to evaluate whether the morphology was suggestive for tuberculous lymphadenitis or not.

Cytological examination of FNA smears were considered diagnostic of TBLN in the presence of the following cytomorphological circumstances: epithelioid cell aggregate with or without Langerhans giant cells and necrosis, epithelioid cell aggregate without necrosis, necrosis without epithelioid cell aggregate or polymorphocytes with necrosis [10].

### Direct smear microscopy for the detection of AFB

Immediately after the specimen was collected, a drop of aspirate was placed on a clean slide to make a direct smear. The standard Ziehl-Neelsen staining procedure was applied [2]. Stained smears were examined for the presence of AFB under oil-immersion (1000x) using a light microscope.

### Concentration method for detection of AFB

FNA specimen was first decontaminated and digested with a 1% NALC-4% sodium hydroxide (NaOH)-2.9% sodium citrate solution and concentrated by centrifugation at 3000 g for 15 minutes at Jimma University Laboratory of Mycobacteriology [2]. After centrifugation the supernatant was decanted carefully and 2–3 drops of the sediment were placed on a clean slide and stained with Ziehl-Neelsen (ZN) acid fast staining. The remaining sediment was used for culture as described below. All AFB smear positive slides were graded based on the IUATLD scale [11].

### Mycobacterial culture

The processed specimens were resuspended by 1 ml of phosphate buffer and inoculated on two LJ tubes. The inoculated LJ tubes were incubated at 37°C and examined daily for the first week for any contamination. For the next 7 weeks LJ media were observed once a week for the growth of mycobacteria. Growth of the mycobacteria was confirmed by visual inspection of colony morphology and microscopic examination of the colonies for acid-fast bacilli (AFB). Differentiation of *M. tuberculosis* complex (MTBc) from non-tuberculous mycobacteria (NTM) was done by para-nitrobenzoic acid susceptibility test at 500µg/ml. A known ATCC strain of H_37_Rv was used as a positive control. Random slants of LJ media were inoculated with sterile distilled water with each run as a negative control.

### Data analysis

Data were analyzed with SPSS version 16.0. Descriptive statistics were used for analysis of socio-demographic and clinical characteristics. Sensitivity, specificity, positive and negative predictive values of diagnostic methods were computed by using culture as gold standard method. A confirmed TBLN case was defined when growth was observed on culture, ZN staining confirmed AFB from the growth and if no growth was observed on LJ media containing PNB.

The association between the efficiency of the test method and factors affecting the efficiency were analysed using binary logistic regression analysis by calculating odds ratios (ORs) with 95% confidence intervals. P-values of <0.05 were considered statistically significant.

### Ethical clearance

Ethical clearance was first obtained from the Jimma University ethical review board. A letter of permission to conduct the study was obtained from Jimma University Specialized Hospital clinical director office. All patients or guardians in case of children were requested for written consent prior to enrolment to the study. Any information concerning the patients was kept confidential. Laboratory results were reported back to the physicians for treatment initiation or decision as early as available.

## Result

### Characteristics of study participants

Thirteen samples from 200 patients with presumptive TBLN that submitted a lymph node aspirate had a contaminated culture result. Thus, only 187 suspects were included in this analysis. Out of 187 suspected patients, 54.4% (102/187) were females. The age range of the suspects was between 3 and 78 years with a mean age of 29.5(±14) years. Half, [49.7% (93/187)], of the study participants were within the 15 to 30 years age group.

The majority (58.3% (109/187)) of clients with presumptive TBLN presented with cervical lymphadenopathy. The mean duration of lymph node enlargement was 11 months. The presence of a lymph node scar was observed in 35.8% (66/187) of cases. Lymph node aspirate appeared purulent in 57.2% (107/187) of the cases. HIV test result was known for only 58.3% (109/187) of suspects; of whom 8.2% (9/109) were HIV positive.

### Detection rate

Out of 187 FNA specimens processed and inoculated on L-J media, *M. tuberculosis* complex was isolated in 68% (127/187) and NTM in 2.1% (4/187) of the cases. Thus, TBLN was confirmed in 68% (127/187) of the suspected cases. On FNAC 79.7% (149/187) of cases were cytomorphologically diagnosed as TBLN. AFB were detected in 25.1% (47/187) of the suspected cases by direct ZN staining and in 49.7% (93/187) of the suspected cases with the concentration method (P<0.0001). The concentration method detected 46 extra patients with an incremental yield of 24.7% (46/187).

### Sensitivity, specificity, positive and negative predictive values

Among 127 culture-positive samples, 44(34.6%) were AFB positive on direct ZN stain and 84(66.0%) on NALC-NaOH concentration method. Out of 56 culture negative samples, only one was positive on direct smear and 7(12.5%) on NALC-NaOH concentration method. Similarly, on FNA cytology 90.5%(115/127) of culture positive cases showed cytomorphological features consistent with TB. From culture negatives, 55.4%(31/56) were classified as TB by FNA cytology. Against culture, direct smear microscopy showed sensitivity of 34.6% and specificity of 98.2%, positive predictive value of 93.6%, and negative predictive value of 39.3%. The NALC-NaOH concentration method had 66.0% sensitivity, 87.5% specificity, 90.5% positive predictive value, and 52.3% negative predictive value. Cytology had sensitivity of 90.5%, specificity of 44.6%, positive predictive value of 77.2%, and negative predictive value of 65.8% (**Table 1**).

**Table 1:** Sensitivity and specificity of direct ZN stain, concentration method and cytology against culture (n = 187).

		Culture		Sensitivity% (95% CI)*	Specificity% (95% CI)
		Positive n(%)	Negative n(%)		
**Direct ZN staining**	Positive (n = 25)	44(97.8)	1(2.2)	34.6%(26.9–43.3)	98.2(90.6–99.7)
	Negative (n = 138)	83(60.1)	55(39.9)		
**Concentration**	Positive (n = 91)	84(92.3)	7(7.7)	66.1(57.6–73.8)	87.5(76.4–93.8)
	Negative (n = 92)	43(46.7)	49(53.3)		
**Cytology**	Positive (n = 146)	115(78.8)	31(21.2)	90.6(84.2–94.5)	44.6(32.4–57.6)
	Negative (n = 37)	12(32.4)	25(67.6)		

*CI = confidence interval.

### Density of acid-fast bacilli (AFB)

On direct ZN method, a total of 47 cases were AFB positive and the majority of them (55.3% (26/47)) were scanty, 29.8% (14/47) cases 1+, 10.6% (5/47) cases 2+ and only 2 cases were graded as 3+. Among 93 AFB positive cases on concentration method, 23.7% (22/93) of cases graded scanty, 43.0% (40/93) were grade 1+, 25.8% (24/93) of cases graded 2+, and 7.5% (7/93) of cases graded 3+.

Among 26 smears which were graded as scanty by the direct method, 14 were increased to 1+ and 9 to 3+ by the concentration method. Of 14 smears graded as 1+ by the direct method, 4 remained 1+ and 9 increased to 2+ by the concentration method (**Table 2**). Among 46 specimens that were AFB positive only after concentration, 47.8% (22/46) had grade of 1+, 41.3% (19/46) cases grade of scanty and 10.9% (5/46) cases grade of 2+.

**Table 2:** Incremental yield of AFB grading on smears prepared after concentration as compared to direct smear microscopy at JUSH, Jimma, South West Ethiopia (n = 47).

Grade on direct ZN method	Grade on concentration method				
	Scanty	1+	2+	3+	Total
Scanty	3	14	9	0	26
1+	0	4	9	1	14
2+	0	0	1	4	5
3+	0	0	0	2	2
Total	3	18	19	7	47

### Cytomorphological features

Hundred forty nine (79.7%) were classified as TBLN on cytology. Out of these, the concentration method detected AFB in 55.7% (83/149) of the cases versus 28.9% (43/149) by direct smear. Similarly, on the culture, mycobacteria were isolated in 77.2% (115/149) of cases diagnosed as TBLN on cytology. The concentration method detected AFB from 10 cases which were missed on cytology: 7 from suppurative abscesses and 3 from pyogenic infections (**Table 3**).

**Table 3:** Comparisons of cytomorphologic features with AFB positivity on direct and concentrated method of Z-N staining (N = 187).

FNAC result	Total cases% (n/N)	Direct ZN staining		Concentrated ZN staining	
		Positive% (n/N)	Negative% (n/N)	Positive% (n/N)	Negative% (n/N)
TBLN	79.7%(149/187)	28.9%(43/149)	71.1%(106/149)	55.7%(83/149)	44.3%(66/149)
Suppurative abscess	7.0%(13/187)	30.8%(4/13)	69.2%(9/13)	53.8%(7/13)	46.2%(6/13)
Pyogenic infection	5.3%(10/187)	0.0%(0/10)	100%(10/10)	30.0%(3/10)	70.0%(7/10)
Reactive LN*	4.3%(8/187)	0.0%(0/8)	100%(8/8)	0.0%(0/8)	100%(8/8)
Other diagnosis	3.7%(7/187)	0.0%(0/7)	100%(7/7)	0.0%(0/7)	100%(7/7)

*Reactive LN = Reactive lymphadenitis.

Detailed cytomorphological features of tuberculous lymphadenitis were clearly described for 82 cases. Out of these, 39.0% (32/82) showed epithelioid cell aggregate with necrosis followed by necrosis without epithelioid cells in 36.6% (30/82), epithelioid cell aggregate without necrosis in 12.2% (10/82) and polymorphocytes with necrosis in 12.2% (10/82) cases.Out of 30 cases with caseous necrosis without epithelioid cells, AFB were present on direct smear in 56.7% (17/30) of them and in 73.3% (22/30) by the concentration method. While of 10 cases showing epithelioid cell aggregates with necrosis, the AFB positivity rate was 1 by direct Z-N staining and 4 by concentration method. AFB positivity both by direct and concentration method increased with the presence of necrosis alone and a lower rate of AFB positivity was observed with the presence of epithelioid cell aggregates alone (**Table 4**).

**Table 4:** Correlation of cytomorphologic features of TBLN with AFB positivity (direct &concentrated smear) and culture positivity (N = 82).

Cytological category	Cases% (n/N)	Direct ZN positive% (n/N)	Concentrated ZN positive% (n/N)	Culture positive% (n/N)
Epithelioid cell with necrosis	39.0%(32/82)	37.5%(12/32)	53.1%(17/32)	68.8%(22/32)
Epithelioid cell without necrosis	12.2%(10/82)	10.0%(1/10)	40.0%(4/10)	70.0%(7/10)
Necrosis without epitheloid cell	36.6%(30/82)	56.7%(17/30)	73.3%(22/30)	86.7%(26/30)
Polymorphs with necrosis	12.2%(10/82)	50.0%(5/10)	60.0%(6/10)	100%(10/10)

**Factors assessed for association with smear positivity on concentration method** **(** **Table 5** **)** Smear positivity by the concentration method was statistically associated with the presence of a lymph node scar [p-value = 0.003, OR = 2.5, 95%CI = 1.3–4.6]. Aspirates with purulent appearance were 2 times more likely to be positive on direct smear microscopy [p-value = 0.037, OR = 2.01, 95%CI = 1.0–4.3]. However, the AFB detection rate with the concentration method did not vary with the nature of the aspirate (p-value = 0.82).

**Table 5:** Factors assessed for associated with smear positivity when considering concentration method alone in Jimma, southwest, Ethiopia.

Factors		Concentration method (ZN)		OR[95%CI]	P-value
		Positive	Negative		
**Sex**	Male	57.6%(49/85)	42.4%(36/82)	1.8[1.0–3.4]	
	Female	43.1%(44/82)	56.9%(58/82)	1.0	0.20
**Age**	0–15	48.1%(13/27)	51.9%(14/27)	1.8[0.5–6.0]	
	15–30	47.3%(44/93)	52.7%(49/93)	1.8[0.7–5.1]	
	30–45	61.7%(29/47)	38.3%(18/47)	3.2[1.1–9.9]	0.51
	>45	35.0%(7/20)	65.0%(13/20)	1.0	
**HIV**	Positive	55.6%(5/9)	44.4%(4/9)	1.1[0.5–1.9]	
	Negative	50.0%(50/100)	50.0%(50/100)	1.0[0.3–4.3]	0.924
	Unknown	48.7%(38/78)	51.3%(40/78)	1.0	
**Lymph node site**	Cervical	49.5%(54/109)	50.5%(55/109)	0.97[0.30–3.06]	0.600
	Axillary	48.6%(18/37)	51.4%(19/37)	1.00[0.28–3.60]	
	Inguinal	64.3%(9/14)	35.7%(5/14)	2.10[.40–11.3]	
**Duration of LN swelling**	≤5 months	48.7%(55/103)	51.3%(58/103)	1.05[0.37–2.99]	0.360
	6–10 months	62.5%(20/32)	37.5%(12/32)	1.88[0.54–6.62]	
	11–15 months	39.1%(9/23)	60.9%(14/23)	0.52[0.14–1.97]	
	>15 months	47.4%(9/19)	52.6%(10/19)	1.00	
**Lymph node mobility**	Yes	52.3%(57/109)	47.7%(52/109)	1.48[0.77–2.83]	0.353
	No	45.3%(34/75)	54.7%(41/75)	1.00	
**Lymph node scar**	Present	63.6%(42/66)	36.4%(24/66)	2.52[1.30–4.89]	0.003
	Absent	42.1%(51/121)	57.9%(70/121)	1.00	
**Gross specimen appearance**	Purulent	50.5%(54/107)	49.5%(53/107)	0.94[0.50–1.76]	0.816
	Caseous	48.8%(39/80)	51.2%(41/80)	1.00	

Of the 9 HIV positive patients, two were positive on direct ZN method and five on concentration method. In HIV infected individuals, the detection rate of ZN staining increased from 22.2% by direct ZN method to 55.6% by concentration method.

## Discussion

In developing countries, like Ethiopia, the conventional diagnostic tool for TBLN mainly relies on FNA cytology and direct smear microscopy. FNAC has limited specificity because of the presence of cytologic components such as epithelioid cells aggregates, multinucleated giant cells, and necrotic material in lesions other than those associated with TB such as fungal infections, other inflammatory conditions and sarcoidosis [5], [6]. Moreover, FNA requires highly trained pathologist which is the case in Ethiopia who are not available at relatively lower level health facilities. Culture is the gold standard for the diagnosis of TBLN. However, the availability and affordability of this method in resource limited settings like Ethiopia necessitates the quest for other techniques with added value over direct Z-N microscopy. In such settings concentrating FNA sample can improve the diagnosis of TBLN.

The low sensitivity (34.6%) of direct smear examination in our study is in agreement with reports from other studies [6], [12]. The nature of specimen and the scanty bacilli found in the lymph node aspirates could be the main factor for the decreased sensitivity of direct smear.

Many reports have shown that liquefaction of clinical samples followed by centrifugation significantly increases the smear sensitivity up to 72% [6], [13]. Our findings demonstrated comparable results. The sensitivity of AFB smear on direct Z-N was 34.6% and it increased to 66.0% on concentration method. The increased smear positivity by the concentration method is attributable to the higher density of bacilli per microscopic field and reduction of debris, leaving a clear field for microscopy.

Seven positive cases on concentration method were culture negative. This may be due to the fact that AFB positivity rate on the concentration method was highest when the cytomorphological feature was caseous necrosis. A crucial phenomenon happens within the caseous lesion is the death of the majority, if not all, of the tubercle bacilli. These non-viable bacilli are unable to grow on culture. Cytomorphological features should be used in conjunction with concentration method to manage such cases in clinical practice.

Our results showed that the majority of positive cases (55.5%) on direct Z-N staining had scanty AFB grade and searching for them was time consuming and tedious. On the concentration method, 76.4% of positive cases showed grades of AFB positivity above scanty, making them easily visible and detectable. Other studies also reported that the AFB positivity grade was higher by the concentration method making the bacilli easily visible within a shorter screening time [6], [13].

AFB positivity rate by direct and concentration method was highest when the cytomorphological features of FNAC was necrosis without epithelioid cell aggregates. By the concentration method, there is an increase in AFB smear positivity from 40.0% in those with epithelioid cell aggregates alone to 53.0% when epithelioid cell aggregates present with necrosis and to 73.3% when necrosis without epithelioid cell aggregates were seen. Similarly, Bezabih *et al.* [14] and Gupta *et al.* [10] observed the highest rate of AFB positivity in smears showing necrosis alone and decreased smear positivity rates with the appearance of epithelioid cell aggregates. This is probably due to the fact that the central necrotic portion of the tubercle contains more bacilli.

Out of 13 cases classified as suppurative abscess on cytology, TBLN was diagnosed in 7 cases by the concentration method and in 4 cases by direct smear microscopy. The possible explanation for the misdiagnosis of specimens as suppurative abscess on cytology may be the absence of characteristic features within abundant mixed inflammatory superinfections by other bacteria.

In the current study TBLN suspects with lymph node scar were 2.5 times more likely to yield positive result on the concentration method as compared to those patients without scar. During infection of lymph node, the caseous material perforates the deep fascia and escapes into the superficial fascia resulting in collar stud abscess formation, the abscess may present with persistent discharging sinus and finally developed in scar.

In conclusion, the sensitivity of the concentration method was significantly higher in comparison with the direct smear microscopy. In addition, the majority of positive cases by the concentration method showed a high grade of AFB positivity, making the screening process easier, faster and less laborious. The highest AFB positivity rate was observed in cytomorphological features consistent with caseous necrosis. Finally we suggested that in addition to routine cytology the concentration method can increase the diagnostic yield for TB lymphadenitis.
